# Hemodialysis with high cutoff membranes improves tissue perfusion in severe sepsis: preliminary data of the Sepsis in Florence sTudy (SIFT)

**DOI:** 10.1186/cc13591

**Published:** 2014-03-17

**Authors:** G Villa, C Chelazzi, S Valente, AR De Gaudio, S Romagnoli

**Affiliations:** 1 University of Florence, Italy

## Introduction

It has been demonstrated that blood purification therapy performed by means of venovenous hemodialysis with high cutoff membranes (HCO-CVVHD) may modulate the host inflammatory response in septic patients with acute kidney injury (AKI), potentially limiting organ dysfunction. Improvement in hemodynamics and respiratory function has been described during HCO-CVVHD treatment [1]. The Sepsis in Florence sTudy (SIFT) has been designed to evaluate changes in inflammatory biomarkers and tissue oxygenation/perfusion indexes in septic ICU patients with AKI during HCO-CVVHD.

## Methods

Patients with microbiologically confirmed severe sepsis/ septic shock and AKI (RIFLE criteria F or more) treated with HCO- CVVHD, started within 12 hours from the diagnosis, were prospectively included in the study. The cumulative vasopressor index (CVI), C-reactive protein levels (CRP), serum lactate concentration (Lac) and central venous oxygen saturation (ScvO_2_) were measured before (T0h) and at 24 hours and 48 hours after HCO-CVVHD initiation. Data are expressed as the median (range). The Mann-Whitney U test was applied to detect differences in CVI, CRP, Lac and ScvO_2 _at the three time points (statistical significance for *P *< 0.05).

## Results

In 16 ICUs, a total of 16 patients (six cardiac surgery, four abdominal surgery and six medical) met the inclusion criteria and were enrolled in the study. A significant reduction in CRP levels was observed over time: 263 (216 to 358) mg/dl at T0h to 153 (56 to 186) mg/dl at T48h *(P <*0.05). ScvO_2 _significantly increased from 45 (40 to 55)% at T0h to 75 (68 to 77)% at T48h (*P *< 0.05). Finally, serum lactate decreased from 5.1 (3.0 to 9.5) mmol/l at T0h to 1.6 (1.0 to 4.6) mmol/l at T48h (*P *< 0.05). Conversely, CVI did not significantly reduce over time (8.2 (4 to 9) at T0h vs. 4.5 (4 to 8) at T48h, *P *>0.05).

## Conclusion

Our preliminary data show that patients with sepsis- related AKI may benefit from early treatment with HCO-CVVHD. The modulation of proinflammatory and anti-inflammatory mediators, as previously demonstrated [1], may improve microcirculation, tissue perfusion and cellular oxygenation. Although promising, our results must be confirmed at the end of the study with larger observations. Finally, a subgroup analysis is absolutely mandatory in order to explore different behaviors of tissue perfusion indexes in different populations of patients.
